# Exostose sous-unguéale: à propos d’un cas et revue de la littérature

**DOI:** 10.11604/pamj.2022.43.137.35277

**Published:** 2022-11-14

**Authors:** Mohamed Ben Jemaa, Hedi Chaabouni, Mohamed Ghorbel, Ameur Abid, Wassim Zribi, Mohamed Zribi, Moez Trigui, Kamel Ayedi, Mourad Aoui, Hassib Keskes

**Affiliations:** 1 Service de Chirurgie Orthopédique et Traumatologique, Centre Hospitalier Universitaire Habib Bourguiba Sfax, Sfax, Tunisie

**Keywords:** Exostose, sous-unguéale, gros orteil, cas clinique, Exostosis, subungual, big toe, case report

## Abstract

L´exostose sous-unguéale est une tumeur ostéocartilagineuse bénigne, peu fréquente et à tendance récidivante. Nous rapportons un cas chez un garçon de 17 ans. La notion de traumatisme était retrouvée. Le traitement est chirurgical par un abord direct. La tumeur a été excisée complètement. Les suites opératoires sont simples sans récidive.

## Abstract (en)

Subungual exostosis is a benign, uncommon osteocartilaginous tumour that tends to recur. We here report the case of a 17-year boy with subungual exostosis, who reported a history of trauma. Treatment was based on direct surgery. The tumor was completely excised. The postoperative course was uneventful, with no recurrence identified.

## Introduction

L´exostose sous-unguéale est une tumeur ostéo-cartilagineuse bénigne, peu fréquente et à tendance récidivante, touchant essentiellement le gros orteil. Elle a été décrite en 1847 par Dupuytren qui, à travers une série de 30 cas, a précisé qu´il s´agissait d´une lésion phalangienne et non unguéale [1]. Le traitement est chirurgical et doit préserver l´ongle [2].

## Patient et observation

**Figure 1:**
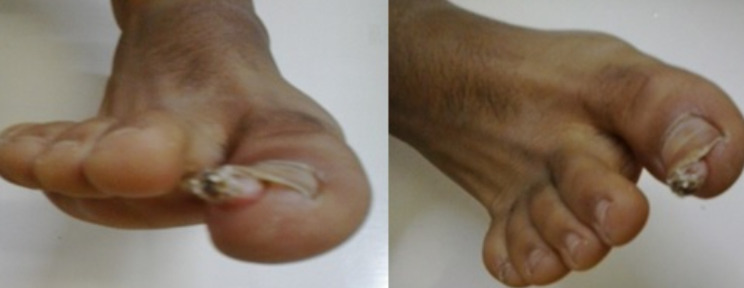
excroissance décollant l´ongle

**Informations relatives aux patients (présentation du patient)**: il s´agissait d´un garçon âgé de 17 ans ayant eu un traumatisme du gros orteil droit deux ans auparavant. Il présentait une excroissance décollant l´ongle évoluant depuis sept mois avec une discrète gêne lors du chaussage sans douleur franche (Figure 1). Cette excroissance était suintante, rebelle au traitement antibiotique et aux soins locaux.

**Résultats cliniques**: l´examen clinique révélait un soulèvement de la tablette unguéale du gros orteil par une excroissance charnue de consistance dure ovoïde faisant 1 cm de diamètre sans signes inflammatoires locaux ni douleur à la palpation, ni signes en faveur d´une onychomycose. L´examen des autres ongles et du reste des téguments était normal.

**Figure 2:**
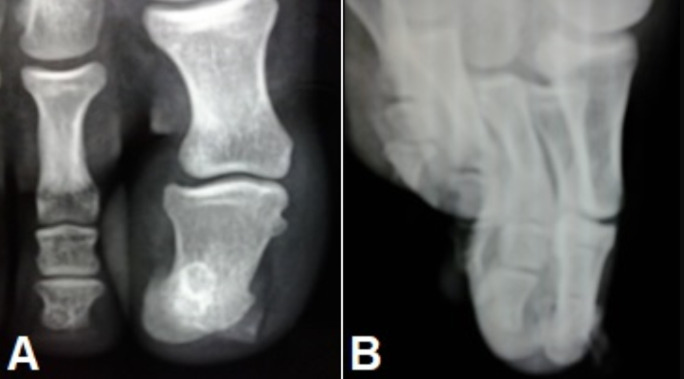
aspects radiologiques de l´exostose sous unguéale; A) incidence de face; B) incidence de profil

**Démarche diagnostique**: la radiographie standard montrait une prolifération osseuse ovoïde à la face dorsale de la houppe de la deuxième phalange de l´hallux (Figure 2). La tomodensitométrie révélait une prolifération osseuse avec matrice cartilagineuse en faveur d´une exostose sous-unguéale.

**Figure 3:**
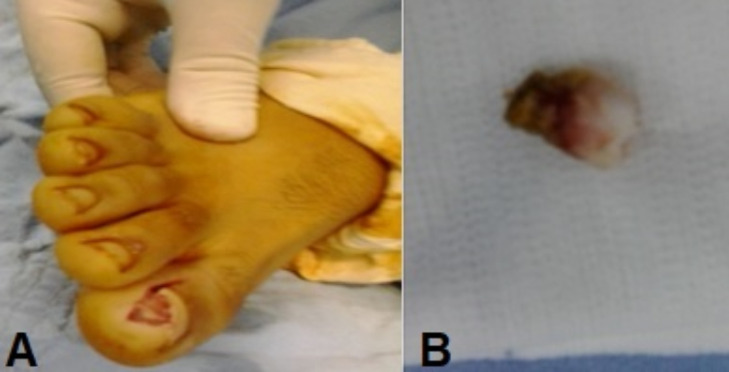
exérèse de l’excroissance osseuse; A) aspect en per-opératoire; B) la pièce de résection

**Intervention thérapeutique**: il a bénéficié d´un traitement chirurgical avec une excision de la partie incarné de l´ongle, une exérèse de l´excroissance osseuse et un curetage soigneux du plan osseux de la phalange distale (Figure 3).

**Figure 4:**
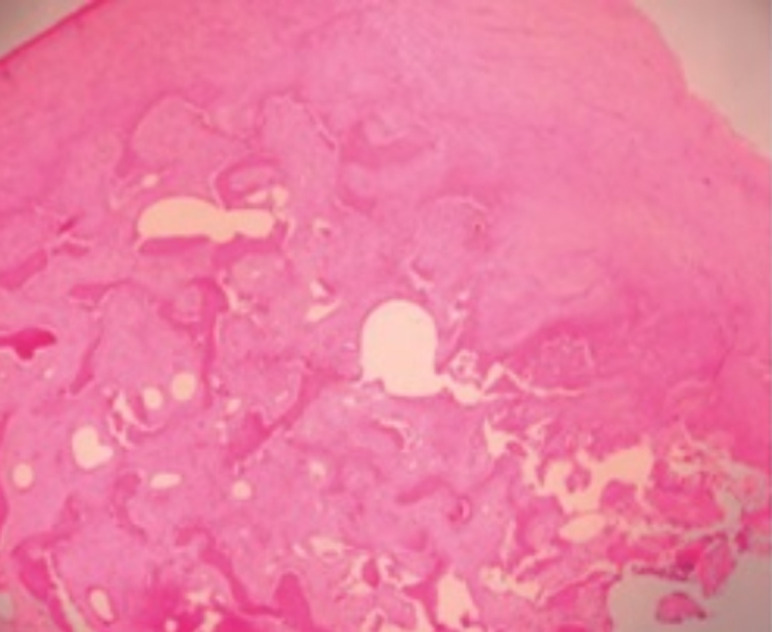
aspects anatomo-pathologiques de l´ostéochondrome

**Suivi et résultats des interventions thérapeutiques**: l´examen anatomo-pathologique a conclu à un ostéochondrome avec des travées osseuses avec remaniement fibreux inter-trabéculaire, recouvertes par une coiffe cartilagineuse irrégulière (Figure 4). Les suites opératoires ont été simples avec reprise de la scolarité, de l´activité sportive et sans aucune gêne au chaussage.

**Consentement**: le patient a été bien informé des données et des objectifs de la publication. Un consentement verbal et écrit a été obtenu.

## Discussion

L´exostose sous-unguéale (ESU) est une entité pathologique rare. Elle touche aussi bien l´homme que la femme avec atteinte prédominante peut se rencontrer de l´enfant et de l´adulte jeune [1,3,4]. Le gros orteil est le siège électif au niveau du pied. L´atteinte de la main est plus rare [3]. L´étiologie de cette maladie reste encore inconnue. Quelques théories ont été rapportées dans la littérature: **la théorie traumatique ou microtraumatique**: l´ossification de l´hématome péri-fracturaire de la deuxième phalange du gros orteil peut engendrer une véritable ESU. Les microtraumatismes répétitifs fréquemment trouvés chez les sportifs de haut niveau (footballeurs) et les personnes portant des chaussures étroites provoquent une irritation osseuse à l´origine d´une prolifération osseuse. L´ESU peut être considérée comme une ossification d´une métaplasie cartilagineuse favorisée par une irritation de l´os [5]; **la théorie tératologique**: selon Williams, l´ESU pourrait provenir d´un os résiduel rudimentaire: le préhallux [3]; **la théorie infectieuse**: le rôle de l´infection dans cette lésion est douteux; **la théorie héréditaire**: plusieurs cas d´ESU ont été décrit dans le cadre d´une maladie exostosante multiple qui représente une maladie héréditaire autosomique dominante [1,4-7]. Dans notre cas, bien que la notion de traumatisme était notée dans les antécédents du patient, il faut penser à la maladie exostosante et chercher d´autres exostoses notamment dans leur localisation profonde du squelette axial. Sur le plan clinique, il s´agit d´une tuméfaction en regard de la phalange distale soulevant l´ongle. Elle peut être ulcérée et/ou surinfectée. L´atteinte du pied et en particulier du gros orteil est la plus fréquente [3,7]. La tablette unguéale soulevée entraine un conflit très douloureux au chaussage avec risque de surinfection amenant les patients à consulter en orthopédie ou en dermatologie [2,8]. Nous pensons qu´il faut évoquer de principe une ESU devant tout ongle incarné avec surinfection récidivante et demander une radiographie standard pour éliminer une ESU sous-jacente. La radiologie permet d´établir le diagnostic d´une ESU en visualisant une excroissance ostéo-condensante bien circonscrite en continuité avec l´extrémité distale de la dernière phalange du gros orteil sans ostéolyse ni autres anomalies osseuses. Dans les formes immatures encore cartilagineuses, la radiographie est difficile à interpréter. Elle peut objectiver montre une image dense sans continuité à la phalange. L´IRM trouve ici son indication. L´exostose sous-unguéale pose un problème de diagnostic différentiel avec le chondrome phalangien, la tumeur glomique sous-unguéale, la verrue sous-unguéale, le mélanome sous-unguéal, le fibrome sous-unguéal, le granulome à corps étranger et l´inclusion épidermique sous-unguéale [1,3,6,7].

Sur le plan histologique, l´exostose sous-unguéale est une lésion ostéo-cartilagineuse similaire à l´ostéochondrome développée au sein d´une phalange distale. Elle comprend trois zones différentes: une coiffe cartilagineuse souvent recouverte d´une lame fibreuse et composée de chondrocytes, une partie centrale formée d´os spongieux et une base d´implantation en continuité avec la corticale de l´os sous-jacent. Le traitement de l´ESU est chirurgical consistant en une exérèse complète de cette excroissance osseuse avec avivement du lit osseux sous-jacent pour prévenir le risque de récidive. L´ongle doit être protégé des lésions iatrogènes du lit et de la matrice, exposant au risque de dystrophie ou de retard de cicatrisation. L´abord direct est utilisé dans les lésions sous-unguéales de localisation centrale Il consiste en un abord longitudinal du lit de l´ongle avec avulsion partielle ou totale de l´ongle. Il expose largement la tumeur et diminue ainsi le risque de récidive mais entraine des lésions de dystrophie unguéale par atteinte la matrice. D´autres auteurs préfèrent un abord latéro-unguéal pour protéger cette matrice [2,9,10]. Chez notre patient, l´excision de la partie incarnée de l´ongle a permis de donner accès direct à la tumeur et de traiter l´ongle malade.

## Conclusion

L´exostose sous-unguéale est une entité pathologique rare. Elle siège touche préférentiellement le gros orteil. Elle doit être suspectée devant toute douleur unguéale au chaussage ou une déformation unguéale. Seule une exérèse chirurgical complète est le garant de meilleurs résultats thérapeutiques avec absence de récidive tumorale.
